# Supplementary material for: Integrated evaluation of lung disease in single animals
Source: PLoS One. 2021 Jul 8;16(7):e0246270. doi: 10.1371/journal.pone.0246270 (PMC8266100; doi:10.1371/journal.pone.0246270)
Supplement: S1 Fig — (A) Histology following H&E stain (scale bar = 100 μm, 20X magnification on camera) from sham-treated (upper panel) or Pseudomonas-infected (lower panel) WT at 24 hours post (hp) treatment (t) or infection (i). Two representative images for sham and three presentative images for infected groups are shown. (DOCX) [file pone.0246270.s001.docx]

**
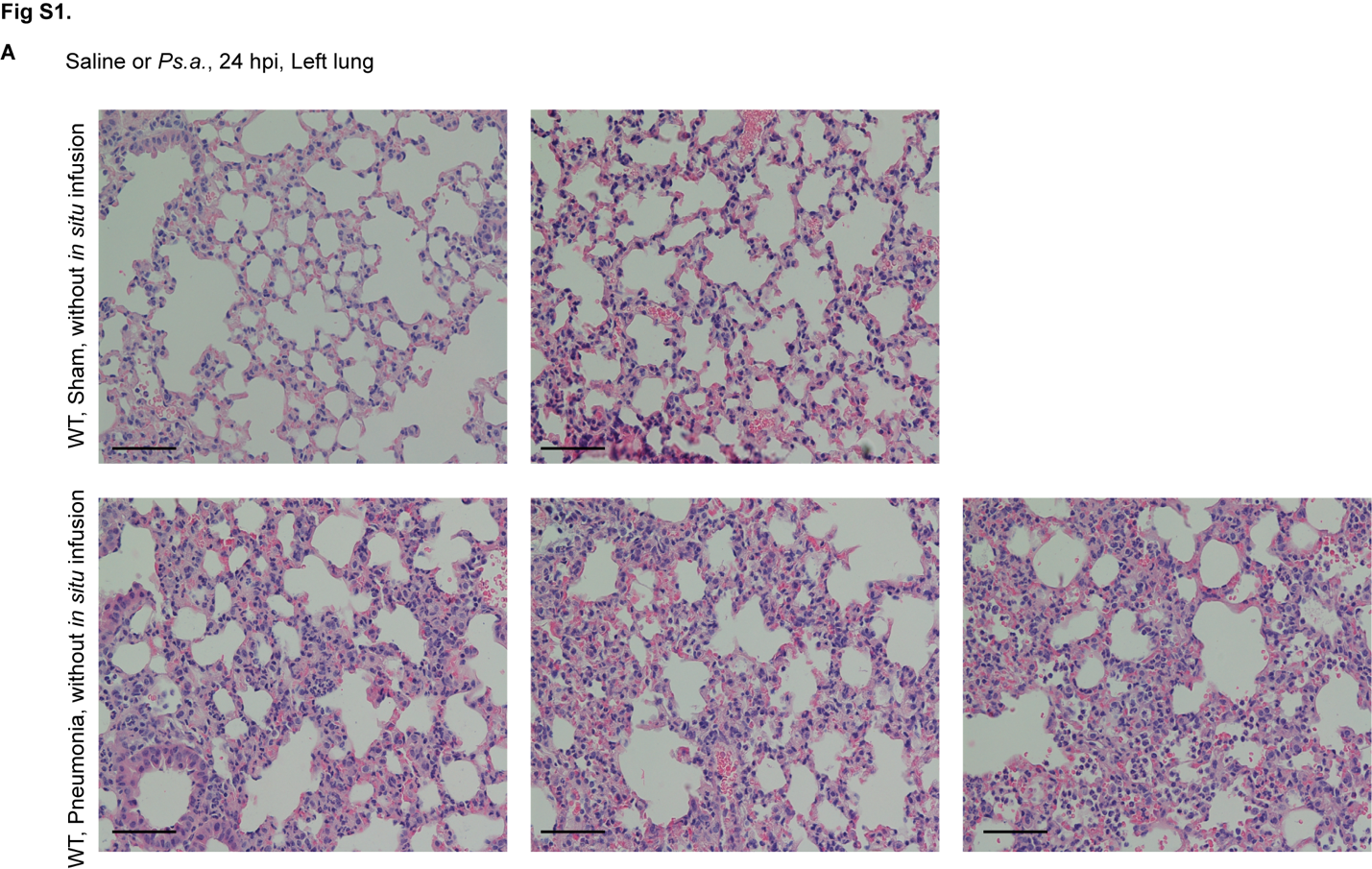
**

**S1 Fig. (A)** Histology following H&E stain (scale bar = 100 μm, 20X magnification on camera) from sham-treated (upper panel) or *Pseudomonas*-infected (lower panel) WT at 24 hours post (hp) treatment (t) or infection (i). Two representative images for sham and three presentative images for infected groups are shown.
