# Supplementary material for: Integrated evaluation of lung disease in single animals
Source: PLoS One. 2021 Jul 8;16(7):e0246270. doi: 10.1371/journal.pone.0246270 (PMC8266100; doi:10.1371/journal.pone.0246270)
Supplement: S1 File — (DOCX) [file pone.0246270.s002.docx]

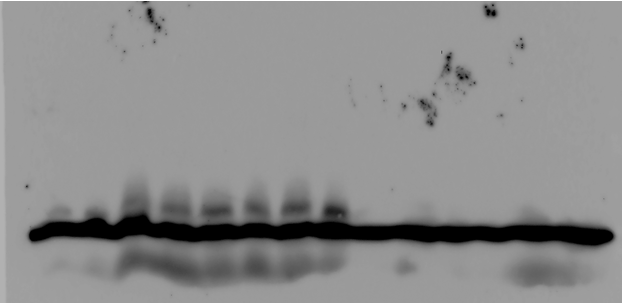

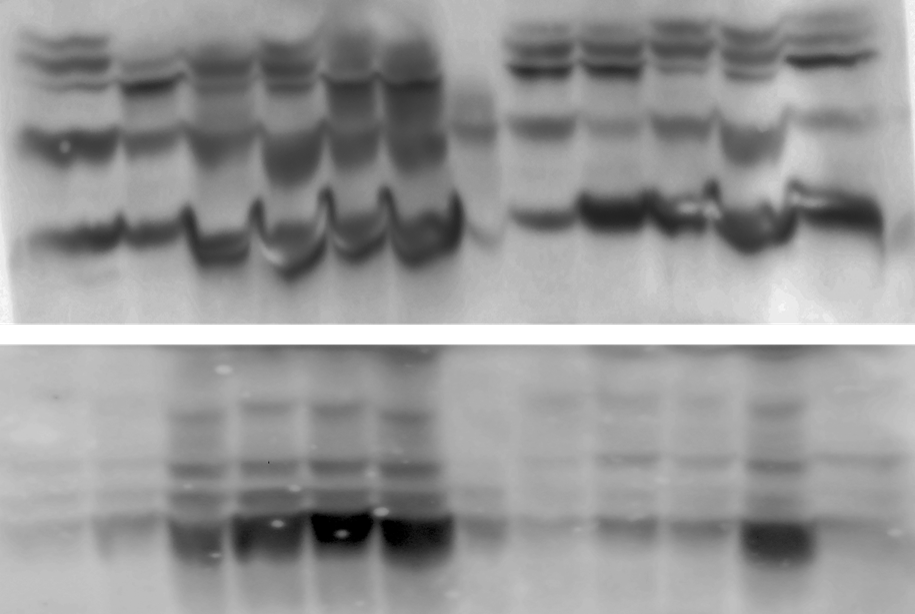
Figure 3D, gels developed on Kwik Quant Gel Imaging System

MW Ladder kDa (Bio-Rad, Kaleidoscope)

12 hpi (shown in manuscript) 24 hpi (not shown in manuscript)

Sham Pneumonia Sham Pneumonia

β-Actin

Cl-C3

Cl-C8

37

25

37

25

20

20

37
