# Supplementary material for: Integrated evaluation of lung disease in single animals
Source: PLoS One. 2021 Jul 8;16(7):e0246270. doi: 10.1371/journal.pone.0246270 (PMC8266100; doi:10.1371/journal.pone.0246270)
Supplement: Attachment — Submitted filename: Response to reviewers 12-1-20.docx [file pone.0246270.s003.docx]

ACADEMIC EDITOR: In addition to addressing the reviewers' concerns on the manuscript, the authors should discuss the limitations of the approach in this study. For example, various compartment of the lungs are not identical either functionally or structurally. Further, some infectious diseases such as TB has been documented to prefer the apical side of the lung. How these aspects would be addressed in your model system needs to be explained in the Discussion section.

Response: We appreciate the review and the editor’s note. We have modified text to clearly indicate how the lung structure varies, as well as limitations of our techniques. The modified Discussion now addresses the editor’s suggestions regarding the processes that take place in apical and basolateral epithelium of the lung.

1. Is the manuscript technically sound, and do the data support the conclusions?

The manuscript must describe a technically sound piece of scientific research with data that supports the conclusions. Experiments must have been conducted rigorously, with appropriate controls, replication, and sample sizes. The conclusions must be drawn appropriately based on the data presented.

Reviewer #1: Yes

Reviewer #2: No

Response: To address the concern by reviewer 2, we have revised the manuscript text so that conclusions are drawn from the data. Statistical comparisons are now included wherever applicable along with sample size information.

2. Has the statistical analysis been performed appropriately and rigorously?

Reviewer #1: Yes

Reviewer #2: I Don't Know

Response: In this modified manuscript, we added statistical comparisons wherever applicable along with sample size information.

3. Have the authors made all data underlying the findings in their manuscript fully available?

The [PLOS Data policy](https://nam03.safelinks.protection.outlook.com/?url=http%3A%2F%2Fwww.plosone.org%2Fstatic%2Fpolicies.action%23sharing&data=02%7C01%7Cpratyusha.mandal%40emory.edu%7Ce2924b4e7f6c4b528b1b08d82e4e2aae%7Ce004fb9cb0a4424fbcd0322606d5df38%7C0%7C0%7C637310261425866595&sdata=8GzGla0yDXCnFgR%2Fj1kqfcETWo6Eb7E8t235TN%2FusTw%3D&reserved=0) requires authors to make all data underlying the findings described in their manuscript fully available without restriction, with rare exception (please refer to the Data Availability Statement in the manuscript PDF file). The data should be provided as part of the manuscript or its supporting information, or deposited to a public repository. For example, in addition to summary statistics, the data points behind means, medians and variance measures should be available. If there are restrictions on publicly sharing data—e.g. participant privacy or use of data from a third party—those must be specified.

Reviewer #1: Yes

Reviewer #2: Yes

Response: We have included all data for all the figures as part of the manuscript.

4. Is the manuscript presented in an intelligible fashion and written in standard English?

PLOS ONE does not copyedit accepted manuscripts, so the language in submitted articles must be clear, correct, and unambiguous. Any typographical or grammatical errors should be corrected at revision, so please note any specific errors here.

Reviewer #1: Yes

Reviewer #2: No

Response: We have corrected all the errors.

5. Review Comments to the Author

Please use the space provided to explain your answers to the questions above. You may also include additional comments for the author, including concerns about dual publication, research ethics, or publication ethics. (Please upload your review as an attachment if it exceeds 20,000 characters)

Reviewer #1: Authors developed a nice technique to minimize the mouse usage in our in vivo experiments and clearly shown the difference between in situ fixation and normal fixation in their IHC studies. However authors mentioned that the in situ fixation process requires 20 minutes with flow through of 10 mL NBF, and authors failed to explain how they protect the other lung from flow through 10%NBF?. I would recommend the authors to include the text that address my concern.

Response: We have edited the text to clarify that unfixed left lung is excised and maintained cold for biological assays, immediately after tying the wire to prevent formalin flow into this lung. The new Figure 2 (an entirely new figure) includes images using hematoxylin dye (in place of formalin) demonstrating that in this occlusion method, the tie restricts flow to the left lung and dye only passes to the right lung.

Also I would recommend to show H&E staining of the whole lung (in low magnification) comparing in situ fixation and normal fixation, that will increase the strength of this manuscript

Response: We now include 10X, 20X and 40X magnification images of lung histology sections in Figure 4. Our initial submission only included 20X and 40X. The current range was selected to give a broad to magnified view of the lung that still demonstrated the differences of sections with or without *in situ* fixation. These distinctions are not objectively discernable at any magnification lower than 10X.

Reviewer #2: Summary:
The research article describes a protocol and its feasibility in different disease-based studies by minimizing the sacrifice of number of animals by using the same animal for histopathological and biological analysis. However authors have failed to justify the applicability of this potential protocol because of lack of experimental presentations. Manuscript has a lot of grammatical errors, some of which are mentioned in minor comments along with the major concerns below.

Response: We have modified the text and included new data in Figures 2, 3, 4 and S1 to address reviewer concerns. We have also corrected all grammatical errors. Please see answers to individual concerns below.

Major comments:
1. Authors mentioned- “During the 20 minutes of right-sided in situ fixation, we removed the left lung cut it into three equal sections and saved individually for biological assays”. Did authors check for any leakage of formalin in the left lung which was stitched to ensure tissue available for biological assays. There is no data presented in this context to show tissue was not or to what percentage it was affected by the formalin.

Response: New Figure 2 shows images using hematoxylin dye (in place of formalin) demonstrating that in this occlusion method, the tie restricts flow to the left lung and dye only passes to the right lung.

2. Authors mentioned-“We passed a 4-0 silk suture (surgical tie) circumferentially around the trachea, below the catheter insertion. The tied silk suture secured the catheter in place (Figure 1B)”. Did authors observe any back flow of formalin in the upper trachea

Response: A small amount of backflow of formalin to the upper trachea occurs as formalin flows with gravity. However, it is important to note that even though there is back flow to the trachea, formalin-fixation of the upper trachea does not influence analysis of lungs.

3. Did authors try single cell isolation from the left lung. Data on viability could be an interesting aspect to look into.

Response: In new Figure 2, we show single cell viability counts. These data demonstrate that the unfixed left lung harvested in this way does not alter viability. Additionally, in Figure 3, we compare lungs excised from cadavers without manipulation of the lungs or with *in situ* perfusion to demonstrate that our perfusion technique does not alter MHV68 titers and cytokine levels. These data demonstrate that in situ formalin fixation of the right lung does not compromise outcome of biological assays using left lung.

4. Since previous publications have already discussed about the in situ formalin fixation for 20 mins and its advantage over the other methods, authors here should have put more emphases and experimental justifications as to how a suture on the left lung was able to establish healthy tissue recovery for different experiments.

Response: We thank the reviewer for this helpful comment.  *“In situ* formalin fixation of right lung” section under Results now emphasizes that the suture is used to restrict formalin flow. The modified text is quoted below:

“We tied a sterile 4-0 silk suture around the hilum of the left lung (Figure 1C left and zoomed in right panels). This prevents formalin flow into the left lung. The tracheal angiocatheter was connected to IV tubing attached to a ten ml syringe fixed at a height of 20 cm above the cadaver (Figure 1D). This process also elevated the trachea above the level of the lung facilitating flow of formalin.”

Furthermore, “Left lung occlusion” section under Results describes in our observations confirming that the left lung is occluded in our technique.

5. Authors mentioned about the applicability of this method in COVID-19 research as well. But they have not shown any data in that respect. Simple virus infection studies at BSL2 level along with the gram-negative bacteria reported here would have helped in justifying these statements.

Response: We have added the MHV68 data to Figure 3 demonstrating that our described technique can be adapted for lung studies with a BSL2 virus.

Minor comments:
Please go through the typos etc in the manuscript some are mentioned below.
1. Line 123: “midline thought the chest”- correction needed.
2. Line 318: “lung injury will significant aid studies on following” correction needed.
3. Line 337: “both that might be result in results that” correction needed.

Response: We have corrected these errors.

6. PLOS authors have the option to publish the peer review history of their article ([what does this mean?](https://nam03.safelinks.protection.outlook.com/?url=https%3A%2F%2Fjournals.plos.org%2Fplosone%2Fs%2Feditorial-and-peer-review-process%23loc-peer-review-history&data=02%7C01%7Cpratyusha.mandal%40emory.edu%7Ce2924b4e7f6c4b528b1b08d82e4e2aae%7Ce004fb9cb0a4424fbcd0322606d5df38%7C0%7C0%7C637310261425871584&sdata=9rdZzFdmYLRSv8ITqPpW1CNehI6Sa4blRdtPNhJkWWc%3D&reserved=0)). If published, this will include your full peer review and any attached files.


If you choose “no”, your identity will remain anonymous but your review may still be made public.


**Do you want your identity to be public for this peer review?** For information about this choice, including consent withdrawal, please see our [Privacy Policy](https://nam03.safelinks.protection.outlook.com/?url=https%3A%2F%2Fwww.plos.org%2Fprivacy-policy&data=02%7C01%7Cpratyusha.mandal%40emory.edu%7Ce2924b4e7f6c4b528b1b08d82e4e2aae%7Ce004fb9cb0a4424fbcd0322606d5df38%7C0%7C0%7C637310261425876573&sdata=JMupI6aj4j46pIr8h8WlADlwS9NW8PaSc7%2B%2FM3m5154%3D&reserved=0).

Reviewer #1: **Yes:** Murugesan Rajaram

Reviewer #2: No

[NOTE: If reviewer comments were submitted as an attachment file, they will be attached to this email and accessible via the submission site. Please log into your account, locate the manuscript record, and check for the action link "View Attachments". If this link does not appear, there are no attachment files.]

While revising your submission, please upload your figure files to the Preflight Analysis and Conversion Engine (PACE) digital diagnostic tool, [https://pacev2.apexcovantage.com/](https://nam03.safelinks.protection.outlook.com/?url=https%3A%2F%2Fpacev2.apexcovantage.com%2F&data=02%7C01%7Cpratyusha.mandal%40emory.edu%7Ce2924b4e7f6c4b528b1b08d82e4e2aae%7Ce004fb9cb0a4424fbcd0322606d5df38%7C0%7C0%7C637310261425881565&sdata=shTsOC5dOgLcMcLuvJn9CWD51PqSns1wLg6lWIs5sI8%3D&reserved=0). PACE helps ensure that figures meet PLOS requirements. To use PACE, you must first register as a user. Registration is free. Then, login and navigate to the UPLOAD tab, where you will find detailed instructions on how to use the tool. If you encounter any issues or have any questions when using PACE, please email PLOS at [figures@plos.org](mailto:figures@plos.org). Please note that Supporting Information files do not need this step.
