# Supplementary material for: Integrated evaluation of lung disease in single animals
Source: PLoS One. 2021 Jul 8;16(7):e0246270. doi: 10.1371/journal.pone.0246270 (PMC8266100; doi:10.1371/journal.pone.0246270)
Supplement: Attachment — Submitted filename: Rebuttal Letter.docx [file pone.0246270.s004.docx]

ACADEMIC EDITOR:

Figure 1: all panels should be labelled. Right now, the “zoom-out” image at far right doesn’t have any label. Correct the legend accordingly.

As commented by Reviewer#2, the legend for Fig4A and Fig S1A have the scale bars wrongly typed. Correct this information.

Line 470. What is 12hp ?. The manuscript quality would improve if such unusual abbreviations are avoided throughout.

Response: We have addressed all of the concerns in the manuscript. We have labeled and corrected corresponding legend for “zoom-out” image in Fig1. We have legends for Fig4A and FigS1A to indicate the correct measurement in μm. We have corrected abbreviations and checked manuscripts to avoid unusual abbreviations.

REVIEWER CONCERS:

Reviewer #3: Authors addressed all questions raised. I have a few more concern.
Authors did not mention which statistical test they used for the data analysis in statistical analysis section.
There is typo in legend of the figure 4. Scale bar is given 200 mm, 100 mm and 40 mm for different panels. However, authors did not mentioned magnification of images respective to each panel in methodology section as well as in figure legend. I think scale bar should be presented in μm instead of mm.

Response: We have now included in the statistical analysis section a complete description of the statistical test that was used for data analysis.

We thank the reviewer for catching the error in typing where scale bars are mentioned. We have corrected it for both Fig4 and FigS1A. We also indicate in both legends what lens magnification of the microscope used each scale bar corresponds to.
